# Effect of reduced-calcium and high-calcium cheddar cheese consumption on the excretion of faecal fat: a 2-week cross-over dietary intervention study

**DOI:** 10.1007/s00394-023-03118-8

**Published:** 2023-02-23

**Authors:** Emma L. Feeney, Aisling Daly, Simone Dunne, Victoria Dible, Rebecca Barron, Sanja Seratlic, J. C. Jacquier, Michael O’Sullivan, Tom Beresford, Søren Krogh Jensen, Eileen R. Gibney

**Affiliations:** 1 UCD Institute of Food and Health, Science Centre South, Belfield, Dublin 4, Ireland; 2 Teagasc Food Research Centre, Moorepark, Fermoy, P61 C996 Co. Cork Ireland; 3 Department of Animal Science, Aarhus University, Blichers Allé 20, Postboks 50, 8830 Tjele, Denmark

**Keywords:** Cheese matrix, Dairy matrix, LDL-cholesterol, Faecal fat excretion

## Abstract

**Purpose:**

Studies show that dairy fat consumed in the form of cheese reduce LDL-cholesterol concentration (LDL-c) compared to butter and mechanistic suggestions include the calcium content of cheese leading to enhanced faecal fat excretion. The aim of this study was to test the effect of varying the calcium content within a cheese, on faecal fat excretion as a primary outcome, and blood lipid markers, fasting glucose and calcium excretion as secondary outcomes.

**Methods:**

7 healthy males (BMI 18–25) participated in this randomized, cross-over control intervention, of 3 × 2 week periods. Diets contained 240 g/day cheese; a High Calcium Cheese (HCC) diet, a Reduced Calcium Cheese (RCC) diet, and a control arm: Reduced Calcium Cheese + CaCO_3_ Supplement (RCC + Supp) diet. Diets differed in calcium content and form but were otherwise controlled for energy and key macronutrients. Blood and 5-day faecal samples were collected.

**Results:**

There was no significant difference in faecal fat excretion (g/day) between the diets (*P* = 0.066). Percent fat of faecel excretion was higher after RCC + Supp (*P* = 0.016). None of the individual fatty acids were different. Fasting LDL-c was significantly lower following the HCC diet vs. the other arms (*P* = 0.002). Faecal Ca was different across all diets (*P* = 0.001), lowest after RCC, and greatest after RCC + Supp. No differences were observed for fasting blood parameters or changes in anthropometry.

**Conclusion:**

Varying the calcium content within a cheese matrix significantly affected fasting LDL-c values. Results did not support higher faecal fat excretion as an underlying mechanism, but the high attrition rate was a limitation.

*Trial registerer* Trial Registered at ISRCTN.org, registration number ISRCTN11663659 on 12.07.2022. Retrospectively registered.

**Supplementary Information:**

The online version contains supplementary material available at 10.1007/s00394-023-03118-8.

## Introduction

There is growing evidence that total fat, and saturated fat, consumed in different food matrices has different cardiometabolic and other health outcomes [1, 2]. The food matrix effect describes the interaction of the overall food structure and how the nutrients contained within, may differentially impact digestion and absorption [3]. The dairy food matrix is a particular example of this, with results from a number of randomized controlled trials and observational studies showing that fat from cheese consumption compared to butter is associated with differences in blood lipid profiles, whereby low-density lipoprotein-cholesterol (LDL-c) concentrations [4–6] or total cholesterol [7] were lower following cheese intake compared to butter.

A 2015 meta-analysis of the available randomised controlled trials calculated that a weighted mean difference of 145 g (cheese vs. butter) resulted in a 6.5% reduction in LDL-c concentration [8]. Various mechanistic reasons for these differences have been postulated. These include the protein content of cheese [9, 10], the fermentation which may modify the gut microbiota [9], greater phospholipid content [11], and greater mineral (Ca and P) content of cheese [10] vs. butter. It is likely that all of these contribute to some degree, but data on these individual parameters is lacking to date, mostly as it is difficult to test the various hypothesis in a single study and or modify a single component within an experimental approach without impacting the other aspects.

With respect to the mineral content, particular emphasis has been given to dairy calcium [12–14]. It is suggested that during digestion, this may interact with the fat in cheese to form insoluble calcium soaps, which in turn are excreted, resulting in reduced intestinal fat absorption (and, therefore, increased faecal fat excretion [12, 15]. Dairy calcium may also complex with phosphate to form CaP, which can bind intestinal bile acids, leading to their excretion in faeces, and resulting in the use of circulating cholesterol for do-novo synthesis of BA, subsequently lowering circulating cholesterol [16, 17].

While several studies to date have suggested that dairy calcium is associated with reduced LDL-c concentration [12, 13], or triglycerides [18], studies on fat excretion have not been conclusive. Dairy Ca was shown to increase faecal fat excretion in some studies [13, 14], but not significantly so in others [18]. However, these studies have used a variety of dairy foods, each with different matrices, which may have confounded the interpretation. For example, Soerensen and colleagues examined milk vs. cheese, while the study by Bendsen et al., provided the dairy fat from butter, and the Ca from a variety of low-fat dairy products. Few have examined Ca in cheese alone. Previously, we observed a significant matrix effect of cheese on blood lipids following a 6 week intervention study, controlling for other macronutrients and calcium [6]. Here, we were interested specifically in the effect of adjusting the calcium within the cheese matrix itself. The aim of this study, therefore, was to examine the impact of additional dairy calcium contained within the matrix of cheese (with a naturally enhanced Ca cheese), and outside of the matrix, using a naturally reduced Ca cheese plus a supplement, on faecal fat excretion (FFE). We hypothesized that FEE would be greater in the HCC and HCC + Supp diets compared to the RCC diet.

## Materials and methods

### Study design and intervention

The study was a randomized, cross-over control design, consisting of 3 × 2-week periods with a 2-week washout period, in a free-living cohort, designed to test the effect of varying the intake of calcium within cheese during each intervention period. Participants were randomly assigned to a sequence for the three cross-over arms, and were provided with all their food (including the experimental cheeses), and water (to ensure balanced calcium intake) for each 2-week intervention period. The three diets during these arms contained 240 g daily of cheese; a High Calcium Cheese (HCC) diet, a Reduced Calcium Cheese (RCC) diet, and a control arm, consisting of the same Reduced Calcium Cheese + CaCO3 Supplement (RCC + Supp), to match the calcium content of the High Calcium Cheese diet (See Table 1). 240 g of cheese was required to achieve the difference in Ca between the diets. The diets were calculated based on average requirements for a healthy 70 kg male, for a moderate physical activity level (PAL) of 1.76 for males 18–30, based on Henry (2005) [19]. In brief, a number of menus were created to offer a breakfast, lunch and main meal for each day, plus some snacks. Cheese was to be incorporated at each meal and snack, spread throughout the day. The menus were balanced for macronutrient content and calcium content. Volunteers selected a menu or menus according to their preferences, and food was collected every 4 days from the human intervention suites at UCD. Volunteers were provided with the same foods (differing only in the cheeses) during each 2-week experimental period. Full details of the menu foods provided are available in Supplemental Table 1. Diets were matched for total energy, macronutrients and fat, and differed in the amount and form of Ca provided. The three diets provided 12.62 MJ per day on average,  ± 0.07 MJ and excluding the cheeses, provided 8.59 MJ per day on average, ± 0.12 MJ (Table 1).

**Table 1:** An overview of the macronutrient content provided by the 3 diets

Nutrient	Food only (cheese and supplement excluded)	High-calcium cheese diet (cheese and food combined)	Reduced calcium cheese diet (cheese and food combined)	Reduced calcium cheese diet + supplement (cheese and food combined)
Energy/MJ	8.59	12.7	12.6	12.6
Protein/g	86.2	147.9	143.6	143.6
Carbohydrate/g	249.1	249.1	249.1	249.1
Fat/g	72.4	149.2	147.3	147.3
SFA/g	19.7	65.8	64.6	64.6
Calcium/mg	811.1	2963.9	2143.1	2964.1
Fibre/g	28.8	28.8	28.8	28.8

The mean daily nutrients provided from the experimental menus, without the experimental cheeses are also shown, for reference. SDs values are not available for each diet period, since values are based on the same food menu (shown in ‘Food Only’) for each diet, with the different cheeses added. Values were calculated from the nutrition information provided from the food packaging (tesco.ie) for the macronutrients, and calcium content was estimated using Nutritics software

Participants were also asked to refrain from alcohol, and from other dairy foods during the 2-week study periods, and to eat the cheese without heating or melting, as per a previous study [6], since it remains unknown how melting may affect the matrix [3]. To limit potential calcium intake through different water sources, participants were provided with standardized bottled water (10 mg Ca/L). Participants could consume tea and coffee ad libitum but were requested to drink it without milk and to use the water provided. Participants kept a daily food and beverage compliance record during the study, and recorded any deviations from the study diet provided, their tea/coffee consumption and any other foods they may have eaten. To facilitate cohesive participant engagement and tracking, an online engagement platform (3rd Pillar Clinical), was used to help monitor their progress through each 2-week protocol, with reminders for upcoming sample collections and daily reminders for diary completion. Participants had to respond to each message to confirm they had followed the protocol for each particular day (eaten food, collected stool sample, etc.). Any deviations were recorded in their compliance log.

### Participants

**Fig. 1:**
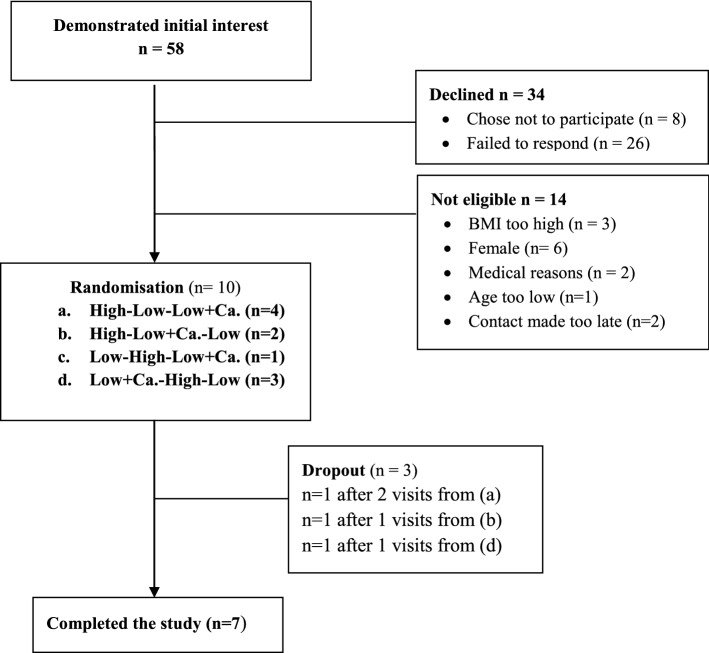
Study flowchart diagram of eligible subjects, randomly assigned. There were *n* = 3 dropouts, due to demanding protocol (*n* = 1), while *n* = 2 were simply lost to follow-up

10 participants were recruited from the general population in Dublin, Ireland, through posters, social media and word of mouth, to participate in the study between May 2017 and May 2018. Inclusion criteria were: male, aged 18–35 years, with a BMI of 18–25 kg/m^2^, and reportedly healthy, with no dairy intolerance or allergy. Exclusion criteria were: any prescribed medication, lactose or milk protein or any food intolerance, following a prescribed diet or actively trying to lose weight. Participants were asked to maintain their existing level of physical activity throughout the duration of the study, and to refrain from drinking alcohol during each 2-week study arm. Participants gave written, informed consent before being enrolled on the study, and all study procedures were reviewed and approved by the UCD Human Research Ethics Committee (LS-16-60-Feeney-Gibney) in accordance with the Declaration of Helsinki. They were not paid to take part but received a One4All voucher at the end of the study, which they received whether they remained to completion or not. A total of seven participants completed all three arms of the intervention. Reasons for dropping out were: demanding protocol and loss to follow-up (i.e. ceased responding) (*n* = 3 in total) (Fig. 1). All biological sample analysis was conducted blind to the intervention.

### Cheese production

Cheeses were produced in Moorepark Technology Limited plant (Moorepark, Co. Cork, Ireland) from milk standardised to a protein-to-fat ratio of 0.92. Briefly, the two cheese varieties with different calcium levels (difference ~ 340 mg/100 g) were produced: one with high-calcium (HC) and one with reduced-calcium (RC) content. The pasteurised milks for HC and RC cheese were inoculated at 31 °C and 28 °C, respectively. The milk for the HC cheese was inoculated at normal milk pH of around 6.7, whereas the milk for the RC cheese was pre-acidified to pH 6.05 with food-grade lactic acid prior to the inoculation of starter cultures comprising of *Lactococci* and *Lactobacilli* strains (Chr. Hansen Ireland Ltd., Cork, Ireland). Rennet (200 IMCU per mL, Chymax Plus, Chr. Hansen Ireland Ltd.) was added after 40 min and the HC and RC milk-gels were cut after approximately 45 and 15 min, then heated to 37 °C and 39 °C, respectively. The RC cheese curd was washed at 34 °C by replacing one third of whey with pasteurised reverse osmosis-treated water, to further deplete Ca from the cheese matrix. The HC and RC whey was drained at pH 6.30 and pH 6.00, respectively, and the curds were cheddared and milled when the pH reached 5.35. The HC and RC curds were salted at a level of 2.8% (w/w), where 40% of salt was replaced with calcium-chloride (Carbon Group, Ringaskiddy, Co. Cork, Ireland) in the HC process. The curds were mellowed for 20 min, moulded (in 23 kg rectangular moulds), pre-pressed at 0.13 kPa for 30 min and pressed overnight at 2.5 kPa at ambient temperature. The following day, cheeses were vacuum-packed and stored at 8 °C for 8 months, and then transferred to 4 °C until consumption.

### Biological measurements

#### Anthropometry

Detailed anthropometric measurements were taken pre- and post-intervention for each 2-week study period. Height was measured with a free-standing stadiometer (SECA). Body weight, BMI and body fat percentage were measured on a Tanita scale (Model BC-420MA), and waist circumference was measured using a standardized measuring tape. Blood pressure was measured using a portable cuff (OMRON M6 Comfort, HEM-7000-E). All measurements were taken in triplicate.

#### Biological samples

##### Blood

Baseline, and post-intervention blood samples (serum and plasma) were collected in the morning following an overnight (12 h) fast, by a trained nurse, using BD Vacutainers containing serum, lithium heparin (LH) and EDTA coagulants. Serum samples were left to coagulate at room temperature for 30 min before processing, whereas EDTA and LH samples were placed on ice and processed immediately. Samples were centrifuged at 4 degrees for 10 min, RCF 1800, RAD 154. Plasma was then aliquoted and stored at -80 until analysis. Total cholesterol, HDL, triglyceride, Non-Esterified Fatty Acid, C-reactive protein and glucose concentrations were determined using a Randox RX Daytona Clinical Chemistry Analyser (Randox Laboratories Ltd, UK). Low and high controls were reconstituted and refrigerated until needed. Before testing began, a reagent calibration was first run, in order to obtain a calibration curve. Samples were thawed at room temperature and then vortexed for 30 s. Controls were included in each run along with a pooled sample, to act as an internal quality check.

##### Urine

24 h urine was collected on the last day of each intervention period. Participants were provided with 2 × 3L containers, 4 × 400 ml containers, ice packs and storage bags. Participants were instructed to discard the first urine sample produced on the morning of day 14 and to collect all urine produced thereafter for 24 h. The samples were kept refrigerated during collection and brought to the UCD Intervention suite on their study visit day (day 15). The total volume of urine produced was recorded. Two 50 ml tubes were filled and placed in the centrifuge at 4 degrees for 10 min (RCF 1500, RAD 154). 10 × 1 ml aliquots were then taken and stored at − 80 until analysis. Urinary calcium was measured using a photometric method via a Cobas 8000- c702 autoanalyser (Roche, UK).

##### Faeces

5-day full faecal samples were collected by participants on the last 5 days of each study period. Participants were provided with airtight sample collection pots (Covidien Commode Specimen Collector, REF2400SA) and were instructed to collect all faecal mass produced over the 5-day period. Samples were kept cool and brought to the UCD laboratory within 24 h of being produced, where they were weighed and stored at − 20. All samples received were freeze-dried, and each 5-day sample (or 4 day and 3 day in some cases) for each participant was pooled and stored at room temperature. Not all seven completers provided full samples on 5 consecutive days. Of the 7 completers, 4 people provided full 5-day samples (12 occasions), while the other volunteers missed a morning or evening sample on 1 or 2 occasions. When this happened, the largest number of consecutive days’ sample was taken and pooled. On three occasions, a pooled 3-day consecutive sample was used, and on five occasions, a pooled 4-day sample was taken. In summary, 13 of the potential 21 5-day pooled samples were achieved for a 5-day full consecutive sample, 5 were for a 4-day sample and 3 were for a 3-day pooled sample. Faecal fat analysis, (and faecal fatty acid profiling) was conducted at the University of Aarhus Denmark, using a modified Bligh and Dyer technique for total fat [16]. FA composition was determined as per Bendsen et al. [14] via fatty acid methyl esters. In brief, the extracted fat was saponified, hydrolysed and methylated. The methyl esters were analyzed using gas chromatography (HP 6890series GC system; Agilent Technologies, Palo Alto, CA, USA), using an automatic on-column injector (HP7673) and a flame ionization detector, with helium as the carrier gas.

### Statistical analysis

The primary study outcome was the difference in faecal fat excretion between the three study intervention arms. The study was powered to observe a minimum of a 25% difference in faecal fat between groups HCC & RCC, based on estimates from other studies [13, 14]. Bendsen et al*.* observed a doubling of faecal fat excretion (from 5.4 g ± 0.5 g d-1 to 11.5 ± 1.4 g) in *n* = 11 participants following a high vs. a low Ca diet (difference of 1600 mg per day), while Soerensen et al*.* observed a 56% difference between FFE between the control diet (500 mg Ca, 3.9 g FFE ± 0.3 g) and a cheese diet (1700 mg Ca, 5.7 g FFE ± 0.4 g), with similar values for the milk diet (1700 mg Ca, 5.2 g FFE, ± 0.4 g). Here, we calculated a sample size of *n* = 8 would be sufficient to observe a difference of 25% (1.25 units of FFE) between the HCC and RCC groups (800 mg) difference, at 80% power with a two-sided 5% significance level, using an estimated ‘within patient’ SD of 0.9. (http://hedwig.mgh.harvard.edu/sample_size/js/js_crossover_quant.html). A target of *n* = 10 participants was set, assuming a 20% dropout rate (aiming for *n* = 8 completers). Unfortunately, due to dropouts (Fig. 1), this target was not quite reached, with *n* = 7 completing the three arms of the study. Since the cheeses were developed and ripened specifically for the study, it was not possible to repeat the trial to increase numbers. Statistical analysis was performed using SPSS version 24 for Mac (SPSS Inc., Chicago, IL, USA). Differences between groups for baseline values (lipids, anthropometry) and mean dietary intakes were tested via one-way ANOVA. Differences between groups for pre- and post-intervention values were assessed using repeated-measures ANOVA, controlling for baseline values, where available (i.e. all blood biochemistry markers). Post hoc analysis was conducted using Fisher’s LSD. Bonferroni correction for multiple tests was applied for individual fatty acid analysis to avoid Type II error, due to the large number of fatty acids. The other outcomes were decided on a priori basis and were not subject to adjustment (Table. 2).

**Table 2:** Nutrient content of the intervention study cheeses and calcium supplement per 100 g

Cheese	Calcium (mg)	Moisture (%)	Protein (%)	Fat (%)	SFA (%)	Energy (kJ)
HCC	897	37.4	25.7	32	19.2	1715
RCC	555	39.4	23.9	31.2	18.7	1665
RCC + Supp^a^	895	39.4	23.9	31.2	18.7	1665

*HCC* high-calcium cheese, *RCC*  reduced-calcium cheese, RCC + Supp = Reduced Calcium cheese, + supplement

^a^Indicates that the calcium value here includes the addition of the calcium supplement, CaCO_3,_ 821 mg Ca total (Holland and Barrett, UK)

## Results

### Dietary intakes

Table 3 shows the average daily intakes during the diet periods, calculated from the food records using Nutritics. There were no differences between the three intervention dietary periods for mean daily macronutrient intake or % energy from these, nor any of the micronutrient intakes (*P* values ranged from 0.213 to 0.827), except for Calcium (*p* < 0.001), which was significantly lower during the RCC diet period, compared to both the RCC + Supp, and the HCC diet periods. There was no difference between the HCC and RCC + Supp diet periods (Table 3).

**Table 3:** Average daily reported nutrient intakes during the 3 diet periods

	HCC		RCC		RCC + Supp		
	Mean	SD	Mean	SD	Mean	SD	*P*
Energy/MJ	13.0	0.9	13.2	0.4	12.8	1.4	0.761
Protein/g	150.9	7.6	149	5.4	145.9	15.2	0.658
Carbohydrate /g	245.5	26.5	258.3	8.5	247.4	26.3	0.518
Total fat/g	162.5	8.6	163.7	4.6	159.7	19.1	0.827
SFA/g	76.5	3.1	76.2	2.1	74.4	9.5	0.792
Calcium/mg	2882.2^a^	95.0	2104.5^b^	71.5	2780.3^a^	410.2	< 0.001
Fibre/g	24.2	2.4	26.8	4.6	23.8	2.7	0.213

Actual (self-reported) intakes were assessed with daily food records, and nutrients calculated using Nutritics dietary analysis software. HCC (High-calcium cheese period), RCC (Reduced calcium cheese period), and RCC + Supplement (Reduced calcium cheese period + CaCO_3_ supplement). Differences in dietary energy and macronutrients were analysed via one-way ANOVA. Superscript letters indicate significant differences

### Participants

A total of *n* = 7 participants (males) completed the study, and ranged in age from 19 to 32 years, with a mean age of 24.4 years (4.1 SD), and a mean weight of 73.0 kg (9 S.D) and a mean BMI of 23.1 kgm^−2^ (1.6 S.D) (Table 4).

**Table 4:** Participant characteristics at baseline

	*N*	Mean	S. D	Range	
				Lower	Upper
Age (years)	7	26.4	4.1	19.0	32.0
Weight (kg)	7	73.0	9.0	60.6	83.3
Height (cm)	7	177.9	9.4	165.2	188.0
BMI (kgm^2^)	7	23.1	1.6	20.2	24.4
Body fat (%)	7	15.3	3.9	10.4	19.6
Waist circumference (cm)	7	78.7	7.3	72.0	92.0
Systolic blood Pressure (mmHg)	7	112.3	5.5	106.0	120.0
Total cholesterol (mmol/L)	7	4.7	0.9	3.1	5.6
HDL -c (mmol/L)	7	1.5	0.3	1.0	1.9
LDL-c (mmol/L)	7	3.2	1.0	1.7	4.6
Triglycerides (mmol/L)	7	0.7	0.1	0.5	0.9
Glucose (mmol/L)	7	5.3	0.4	4.5	5.8
hsCRP (ng/ml)	7	1.9	2.6	0.2	7.3
NEFA (umol/L)	7	485.7	340.2	90.0	1110.0

Anthropometry at the pre- and post-visits for each 2-week dietary period, along with the calculated changes is outlined in Table 5. No differences were observed between the three dietary periods for weight, body fat % or for waist circumference (Table 5) either at the beginning of the study, nor following the three dietary intervention periods.

**Table 5:** Changes in anthropometry following the three intervention diets

Measure	Diets	*n*	Pre			Post			Change		
			Mean	SD	*P*	Mean	SD	*P*	Mean	SD	*P*
Weight/kg	HCC	7	73.24	9.02	0.958	73.05	8.90	0.998	− 0.19	1.46	0.247
	RCC	7	74.65	9.89		73.35	8.94		− 1.30	1.11	
	RCC + supp	7	74.31	9.26		73.24	8.84		− 1.07	1.20	
Body fat %	HCC	7	15.70	3.88	0.854	15.50	4.59	0.830	− 0.20	1.35	0.376
	RCC	7	16.05	4.29		17.00	5.51		0.94	3.20	
	RCC + supp	7	16.98	4.94		15.74	4.58		− 1.24	2.44	
Waist circumference/cm	HCC	7	78.93	7.19	0.903	76.79	5.97	0.898	− 2.14	2.46	0.419
	RCC	7	78.50	7.78		78.14	4.45		− 0.36	4.38	
	RCC + supp	7	77.29	6.01		77.07	6.64		− 0.21	1.11	

Dietary interventions are: HCC (high-calcium cheese), RCC (Reduced calcium cheese), and RCC + Supplement (Reduced calcium cheese + CaCO_3_ supplement). Differences between intervention arms for anthropometry at pre- (V1) and post-intervention (V2) values were calculated using one-way ANOVA. Differences in change (V2–V1) was assessed using a general linear model, controlling for baseline values

The changes in fasting blood glucose and blood lipid profiles are shown in Table 6. There was no difference in the post-intervention total cholesterol, triglycerides or HDL cholesterol values between the three dietary intervention periods (adjusted for baseline values). Fasting LDL-c concentration was significantly lower following the HCC diet (3.36 mmol/L ± 0.84), compared to both the RCC + Supp diet (3.63 mmol/L ± 0.5) and to the RCC diet (3.52 mmol/L ± 0.8).

**Table 6:** Blood glucose and lipids before and after each intervention period, and faecal fat and urinary calcium values following each dietary intervention

	HCC *n* = 7		RCC *n* = 7		RCC + Supp *n* = 7		*P*
	Mean	SD	Mean	SD	Mean	SD	
Total cholesterol (mmol/L)							
Pre	4.83	0.90	4.75	0.64	4.77	0.72	
Post	4.84	0.65	5.03	0.43	4.87	0.65	0.09
HDL-C (mmol/L)							
Pre	1.48	0.26	1.50	0.28	1.45	0.34	
Post	1.47	0.30	1.40	0.36	1.34	0.26	0.766
LDL-C (mmol/L)							
Pre	3.35	1.10	3.11	0.74	3.32	0.89	
Post	3.36^a^	0.84	3.63^b^	0.50	3.52^b^	0.80	**0.002**
Cholesterol ratio (TC:HDL)							
Pre	3.39	1.16	3.29	1.00	3.48	1.08	
Post	3.44	1.08	3.76	0.93	3.76	1.00	0.078
Triglycerides (mmol/L)							
Pre	0.75	0.30	0.81	0.27	0.74	0.19	
Post	0.63	0.17	0.66	0.20	0.63	0.07	0.889
Glucose (mmol/L)							
Pre	5.32	0.44	5.23	0.19	5.37	0.32	
Post	5.20	0.35	5.36	0.27	5.35	0.40	0.490
hsCRP (ng/ml)							
Pre	3.17	3.77	1.13	1.12	0.59	0.52	
Post	1.35	1.02	1.82	2.08	1.56	1.62	0.394
NEFA (umol/L)							
Pre	525.71	316.96	311.42	109.61	350.0	170.88	
Post	997.14	1548.48	835.71	1317.33	831.42	1227.04	0.204
Faecal fat (%)							
Post	17.05^a^	2.54	16.50^a^	2.77	19.30^b^	1.54	**0.016**
Faecal fat excretion (g/day)							
Post	5.72	1.88	4.91	1.21	4.48	1.84	0.066
Faecal Ca (mg/g)							
Post	68.2 ^a,b^	21.64	57.59^a^	17.8	71.7^b^	20.4	**0.01**
Total faecal excretion (g/day)							
Post	33.15	7.09	30.04	6.83	23.37	9.25	0.0826
Urinary Ca (mmol/L)							
Post	4.43	3.7	3.3	2.31	3.9	1.36	0.656

*n* = 7 participants completed each dietary period. HC (high-calcium cheese period), RC (Reduced Calcium cheese period), and RC + Supplement (Reduced calcium cheese period + CaCO_3_ supplement). Differences between the diet groups post-intervention were assessed using general linear models, adjusting for baseline values. Post hoc comparisons were conducted with Fisher's least significant difference (LSD) test. Significant differences in pairwise comparisons are indicated using superscript letters

There was no difference in faecal fat (expressed as g/day), or in urinary calcium excretion. The % of faecal fat was higher during the RCC + Supp cheese diet period (*P* = 0.016) compared to the other two diet periods. There was no significant difference in total faecal excretion between the diets although there was a trend for lower excretion in the RCC + Supp diet. Faecal excretion is subject to considerable variation, and for this reason, excretion rate is typically expressed as a g/day rate, averaged over a 5-day period, and takes into account daily sample fluctuation. To allow comparison with other reports, faecal fat is expressed in both g/day and as a percentage.

Faecal fat excretion (g/day) did not follow the expected trend, with the greatest excretion during the RCC + Supp diet period, although this was not significant (*P* = 0.066). On the other hand, the faecal fat expressed as % was higher following the RC + Supp diet, which corresponded to a non-significant lower total excretion on this diet (Table 6). Faecal calcium was significantly higher following the HCC and RCC + Supp diets compared to the RCC diet, with the RCC + Supp diet showing the highest excretion rates (71.7 mg ± 20.4, vs. 68 ± 21.64 following the HCC diet) (Table 6).

Mean fasting values and SD for blood glucose and lipids were measured at the beginning (pre) and end (post) of each 14-day dietary period; HCC (high-calcium cheese) period, RCC (reduced-calcium cheese) period, and RCC + Suppl (reduced-calcium cheese + CaCO_3_ supplement) period, for *n* = 7 participants who completed all three arms of the study. *P* values indicate repeated-measures GLM for post-intervention values. Values were baseline-adjusted for all blood biochemical markers. Baseline samples were not available for urine (calcium) or faecal (fat and calcium) samples.

### Fatty acid excretion

The faecal samples were also analysed for a range of fatty acids and their relative amounts as a percentage of the total fat is shown in Supplemental Table 2. Overall, there were no differences observed between the diets for any of the individual fatty acids. Only linoleic acid was different across treatments with a slightly higher excretion following the RCC diet, (*P* = 0.011), but this was not significant after Bonferroni adjustment for multiple tests.

## Discussion

In this study, we aimed to examine the effect of increasing the calcium *within* the cheese matrix on faecal fat excretion, in a group of healthy, free-living males, who consumed a diet of higher calcium or reduced-calcium cheese for two weeks, or a reduced-calcium cheese plus a CaCO_3_ supplement (the control diet), in a three-arm cross-over design. The primary outcome of the study was faecal fat excretion (FFE). We also measured post-intervention faecal and urinary calcium, as well as fasting glucose and lipid profiles and anthropometry pre and post-intervention. The mean daily FFE rates (g/day) here ranged from 4.48 to 5.72 g/day. The lowest excretion (4.48 g/day) was seen during the RCC + Supp period, while FFE was highest following the high Ca cheese diet, although not significantly so (*P* = 0.066). Despite the lower total fat excretion rate, faecal fat % was highest in the RCC + Supp diet, indicating that the lower g/day excretion was due to the lower dry matter excretion overall during this dietary period. It is unclear why total faecal excretion would be affected by the supplement, but other studies have observed similar variations with diets. Soerensen and colleagues (2014), in a cross-over trial with milk and cheese-based diets (which were matched for energy, fat content and fibre in a similar manner to this study), observed that the higher dairy calcium diets resulted in higher dry matter excretion. Lower LDL-c post-intervention in milk and cheese diets vs. the control were observed, indicating an LDL-lowering effect from the dairy calcium. In this study, the LDL-cholesterol concentration was significantly lower post-intervention, following the HCC diet compared to the RCC and to the RCC + Supp diets (Table 6). There were no other significant differences observed in the fasting measures of blood lipids or glucose. The lower LDL-c concentration observed following the HCC diet supports a matrix effect of fat and dairy calcium together in the cheese, since the calcium contents were matched in the RCC + Supp group; thus, the effect appears due to more than simply Ca content alone. The slightly higher FFE rate (albeit not significant) following HC cheese suggests that dairy calcium within the cheese matrix is a driver of this effect. A 2016 in-vitro digestion study of enhanced calcium cheddar cheese [20] demonstrated that additional calcium in the matrix resulted in a harder cheese, and during in vitro digestion, matrix disintegration was found to be slower than the lower calcium cheese, while the rate of lipolysis progressed more quickly in the enhanced cheese. This suggests that the calcium content has a significant impact on the overall physical properties of the matrix and may affect accessibility to nutrients within. Further, the cheeses used here were subject to the same fermentation, yet differences were noted between the diets. This suggests that the effects observed cannot be due solely to the fermentation process either. It should be noted that the amount of cheese used in this study was high (240 g per day) and is greater than the recommended portions for most cheeses, which tend to be in the region of 30 g. The large portions were for the experimental purposes described and should not be considered as a recommended daily intake.

The faecal fat excretion rates observed here (4.48–5.72 g/day) were slightly lower than those reported in Bendsen et al*.* (2008), (5.4 g and 11.5 g/day during two 7-day dietary periods of high and low Ca intake), while Buchowski et al*.* [21], in a 12-week weight loss intervention, reported levels of ranging from 3.8 to 5.9 g/day, which are more aligned with those reported here. Several aspects may have contributed to the higher FFE rates observed by Bendsen and colleagues [14]. In their study, FFE was examined in* n* = 11 overweight participants aged 18–50. Mean daily FE was 5.4 g /day during the low Ca diet (52 mg per MJ of dietary energy) and 11.5 g during the high Ca diet (205 mg per MJ), in a crossover study, and the dietary Ca was provided using low-fat dairy foods. Here, the HCC and RCC diets provided 218 mg per MJ and 160 mg per MJ, respectively (data not shown), meaning that the difference in Ca between the high and low periods was less here than those in the study by Bendsen and colleagues, and the dairy foods here (cheese) contained approx. 34% fat per 100 g of product, so would not be considered ‘low-fat’ dairy. Further, the diets here all consisted of 240 g daily cheese intake, which contributed significant protein (~ 60 g per day, to a mean dietary protein of between 143.6 and 147.9 g/day, or 19.03%—19.44 expressed as % energy from protein). Previous work suggests that increased protein intake can increase intestinal calcium absorption [22, 23], potentially reducing the available calcium to bind to the intestinal fat [19]. The relatively high protein content of the diets here (ranging from 19.03 to 19.44%E on average) may have attenuated differences in FFE between the high and low Ca arms. Jacobsen et al. [24] observed FFE rates of 6 g per day during a low Ca diet (500 mg) of 1 week duration, and at a low protein energy (15%E from protein), which increased to 14.2 g/day during the high Ca intervention (1800 mg Ca), but when the %E from protein was increased to 23%, the FFE was unchanged vs the low Ca, at 5.9 g/day. The protein %E in the present study (ranging from 19.03 to 19.44%E on average) falls between the two values reported by Jacobsen’s study of 15%E and 23%.

However, it should be noted that it is unlikely that the protein content here reduced the availability of Ca for soap formation, since the fat excretion rates observed here, and relatively high Ca excretion, suggest that Ca was still available, and thus does not support the formation of unabsorbed soaps as being solely responsible for the LDL–c lowering, suggested by other studies. In the initial power calculations to estimate the difference between the high and low Ca diets, we had assumed a linear relationship between Ca and fat excretion but had not considered potential effects of protein which could impact that linearity. It should also be noted that we observed lower post-intervention LDL-c in the HCC diet vs the RCC and the RCC + Supp diets, despite a lack of increase in FFE following the HCC diet, which would also indicate this was not solely due to soaps excretion.

In both studies by Bendsen et al*.* [14] and Buchowski et al*.* [21], the participants were classified as overweight/obese, whereas here, the participants in the present study were of normal BMI (18–25 kg/m^−^2). While there was 30% energy restriction in study by Buchowski et al*.,* the food in the Bendsen study was matched to each individual, based on their basal metabolic rate (BMR) and their physical activity level (PAL). Here, they were also matched to needs, but on an averaged basis and to a lower BMI, so energy intakes were more similar to those in the study by Buchowski et al*.*, which may explain why the excretion rates here are also similar. In addition to potential effects of protein, Kjølbæk and colleagues [25] reported that fibre may have a confounding effect on the interaction between Ca and faecal fat However, in that study, the range of fibre content was much greater: 20.2 g in Quartile 1 and 32.5 g in Quartile 3. Here, the fibre content did not differ across the diets, and was generally close to the low end of that reported by Kjølbæk et al*.,* ranging from 23.8 to 26.8 g/day. For this reason, we do not think that fibre was a confounder in this study, but this is an important point to highlight.

Boon et al*.,* [18], observed that 7-day intake of 1200 mg calcium, from a non-dairy source, [supplemental CaCO_3_] in a mixed-sex group of *n* = 10 healthy normal weight subjects, led to decreased serum triglycerides. The same study did not observe an impact on triglycerides of either of 400, 1200 and 2500 mg of dairy calcium, suggesting that the CaCO3 form had a greater impact on triglycerides than the same level of Ca from dairy foods. Serum triglycerides in this study were all slightly lower post-intervention compared to pre-intervention values, but there was no difference between the diets. The CaCO_3_ used here provided approx. 820 mg of non-dairy Ca, but this was consumed in addition to dairy Ca, provided in the RC cheese, i.e. the calcium during the RCC + Supp period was from a mixed source, which could account for the lack of differences observed between the diets for triglycerides vs. other studies.

No differences were observed here in the individual fatty acids, other than linoleic acid (Table S2), but after Bonferroni adjustment of the *P*-value cutoff (0.0013) this was not a significant result. This was not entirely unexpected, since the fat consumed was derived, for a large extent, from dairy fat, and so would have been expected to contain similar levels of fatty acids, and the diets were standardized. Although studies have suggested that SFA may be more affected by calcium in the diet [26, 27], a more recent study by Bendsen et al*.* [14]*,* using dairy foods, suggested that MUFA were more affected by the differences in Ca intake than SFA. We did not observe evidence for differences in the fatty acid excretion here. Nonetheless, this information adds to the literature in this area, since few studies report the faecal fatty acid breakdown following dairy consumption.

A number of strengths and limitations to this study should be noted. Firstly, the higher than expected attrition rate was a major limitation and unfortunately, despite the best efforts of the researchers, it was not possible to extend the study, due to the cheeses being specifically made for this study and aged for 8 months prior to the interventions. Additional limitations include the lack of a habitual calcium intake measure, and that cheeses were not measured for their P content. A measure of habitual Ca would have allowed us to identify any low and high habitual consumers, who potentially might have responded differently in their lipid concentrations. Regarding the P content of the cheeses, calcium phosphate has been shown to reduce LDL-c concentration [16], and cheddar cheeses are high in both Ca and P, which may have had an additional impact on LDL-c concentration over and above Ca alone. A further limitation is that we did not measure physical activity levels during this study, and that individual energy estimates were not calculated. Despite these limitations, a particular strength was the rigorous nature of the data collection, and the controlled dietary intake from those who remained in the study. This followed best practice from previous research, and there was considerable contact with participants to encourage compliance, using an automated messaging system. The specifically produced cheeses, allowing only the calcium to be adjusted within the cheese matrix, while controlling other variables (e.g. starter culture, ripening conditions), and the supply of the drinking water, also contributed to a well-controlled dietary intake in each arm.

## Conclusion

This study demonstrates that diets matched for fat content from cheese, but which differ in the amounts of calcium delivered within the matrix, result in differences in fasting LDL-c values, in a group of healthy males. Increasing the level of Ca within the cheese matrix resulted in significantly lower LCL-c vs. the reduced Ca cheese, and compared to the same RC cheese with matched Ca, delivered as a supplement. The mechanistic reason underlying this is unclear. Our results do not support the increased faecal fat hypothesis, but we acknowledge that the high attrition rate here limits the ability to draw strong conclusions on the mechanism. The increased faecal rates with calcium supplementation warrant further study. While the amounts of cheese eaten here (240 g/day) are greater than would be consumed more generally, these findings have implications for dietary guidelines, and provide further evidence that food matrix effects are an important consideration when studying the effects of nutrient and food intake on health outcomes.

## Supplementary Information

Below is the link to the electronic supplementary material.

Supplementary file1 (DOCX 48 KB)

Supplementary file2 (DOCX 19 KB)

## Data Availability

The data that support the findings of this study are not openly available due to ethical approval restrictions on human data, but are available from the corresponding author upon reasonable request.
