# Supplementary material for: Effect of reduced-calcium and high-calcium cheddar cheese consumption on the excretion of faecal fat: a 2-week cross-over dietary intervention study
Source: Eur J Nutr. 2023 Feb 23;62(4):1755–65. doi: 10.1007/s00394-023-03118-8 (PMC10195742; doi:10.1007/s00394-023-03118-8)
Supplement: Supplementary file 1 — Supplementary file1 (DOCX 48 KB) [file 394_2023_3118_MOESM1_ESM.docx]

| **Menu 1** |  |  |  |  |  |  |  |  |  |
| --- | --- | --- | --- | --- | --- | --- | --- | --- | --- |
| **Food items per estimated portion** | **Portion** | **Portion** | **Energy** | **Protein** | **CHO** | **Fat** | **SFA** | **Calcium** | **Fibre** |
| **Breakfast** | **description** | **g** | **kJ** | **g** | **g** | **g** | **g** | **mg** | **g** |
| Soft rolls (brown) | 2 rolls | 140 | 1589 | 16 | 64 | 5 | 1 | 280 | 6 |
| Butter (Dairygold butter sticks) | 2 pats | 14 | 417 | 0 | 0 | 11 | 7 | 3 | 0 |
| Continental meats | 4 slices | 33 | 451 | 8 | 0 | 8 | 3 | 3 | 0 |
| Orange juice (Tropicana 250ml bottles) | 1 bottle | 250 | 455 | 2 | 22 | 0 | 0 | 25 | 2 |
|  |  |  |  |  |  |  |  |  |  |
| **Food items per estimated portion** | **Portion** | **Portion** | **Energy** | **Protein** | **CHO** | **Fat** | **SFA** | **Calcium** | **Fibre** |
| **Lunch** | **description** | **g** | **kJ** | **g** | **g** | **g** | **g** | **mg** | **g** |
| Bread (Hovis best of both) | 2 slices | 80 | 794 | 8 | 33 | 2 | 0 | 382 | 3 |
| Mayonnaise (Hellmann’s) | thick spread | 14 | 415 | 0 | 0 | 11 | 1 | 1 | 0 |
| Ham (Denny, crumbed) | 2 slices | 40 | 190 | 9 | 1 | 1 | 0 | 2 | 0 |
| Lettuce | 4 leaves | 25 | 12 | 0 | 0 | 0 | 0 | 6 | 0 |
| Tomato (cherry) | 6 tomatoes | 90 | 76 | 1 | 3 | 0 | 0 | 9 | 1 |
| Grapes | fifth of pack | 100 | 278 | 0 | 15 | 0 | 0 | 10 | 1 |
|  |  |  |  |  |  |  |  |  |  |
|  |  |  |  |  |  |  |  |  |  |
| **Food items per estimated portion** | **Portion** | **Portion** | **Energy** | **Protein** | **CHO** | **Fat** | **SFA** | **Calcium** | **Fibre** |
| **Dinner** | **description** | **g** | **kJ** | **g** | **g** | **g** | **g** | **mg** | **g** |
| Chicken curry with Rice (Tesco Classics) | whole portion | 500 | 2185 | 41 | 59 | 12 | 2 | 0 | 7 |
| Lemon cake slices (Tesco) | 1 slice | 29 | 493 | 1 | 16 | 5 | 1 | 12 | 0 |
| **Alternate** |  |  |  |  |  |  |  |  |  |
| Cod Cake (Tesco) | 1 cake | 130 | 800 | 13 | 24 | 4 | 0 | 66 | 2 |
| Potato wedges ( Tesco, oven cook) | half portion | 225 | 1292 | 5 | 58 | 4 | 0 | 29 | 8 |
| Chef selection mixed veg (microwave) | 1 third portion | 117 | 178 | 3 | 5 | 1 | 0 | 50 | 3 |
| Lemon cake slices (Tesco) | 1 slice | 29 | 493 | 1 | 16 | 5 | 1 | 12 | 0 |
|  |  |  |  |  |  |  |  |  |  |
| **Snacks** | **Description** | **g** | **kJ** | **g** | **g** | **g** | **g** | **mg** | **g** |
| Popcorn (Manhatten, 6 pack) | 1 packet | 30 | 165 | 1 | 6 | 2 | 1 | 2 | 5 |
| Clementine | 1 fruit | 60 | 106 | 1 | 5 | 0 | 0 | 15 | 1 |
| Rice cakes (Tesco Lightly salted) | 3 cakes | 21 | 339 | 2 | 17 | 1 | 0 | 3 | 1 |
| Humous (Reduced fat selection) | half pot | 50 | 497 | 5 | 6 | 8 | 1 | 21 | 2 |
| Tomato (cherry) | 7 tomatoes | 80 | 67 | 1 | 2 | 0 | 0 | 8 | 1 |
|  |  |  |  |  |  |  |  |  |  |

**Menu 2**

| **Food items per estimated portion** | **Portion** | **Portion** | **Energy** | **Protein** | **CHO** | **Fat** | **SFA** | **Calcium** | **Fibre** |
| --- | --- | --- | --- | --- | --- | --- | --- | --- | --- |
| **Breakfast** | **description** | **g** | **kJ** | **g** | **g** | **g** | **g** | **mg** | **g** |
| Soda Bread (brown retail) | 2 slices | 80 | 730 | 7 | 35 | 1 | 0 | 106 | 6 |
| Butter (Dairygold butter sticks) | 2 pats | 7 | 209 | 0 | 0 | 6 | 4 | 1 | 0 |
| Eggs (boiled) | 1 egg | 50 | 299 | 7 | 0 | 5 | 1 | 28 | 0 |
| Orange juice (Tropicana 250ml bottles) | 1 bottle | 250 | 455 | 2 | 22 | 0 | 0 | 25 | 2 |
|  |  |  |  |  |  |  |  |  |  |
| **Food items per estimated portion** | **Portion** | **Portion** | **Energy** | **Protein** | **CHO** | **Fat** | **SFA** | **Calcium** | **Fibre** |
| **Lunch** | **description** | **g** | **kJ** | **g** | **g** | **g** | **g** | **mg** | **g** |
| Bread (Hovis best of both) | 2 slices | 80 | 794 | 8 | 33 | 2 | 0 | 382 | 3 |
| Mayonnaise (Hellmann’s) | thick spread | 14 | 415 | 0 | 0 | 11 | 1 | 1 | 0 |
| Chicken (Tesco deli slices) | 2 slices | 66 | 293 | 13 | 1 | 1 | 0 | 6 | 1 |
| Lettuce | 4 leaves | 25 | 12 | 0 | 0 | 0 | 0 | 6 | 0 |
| Tomato (cherry) | 6 tomatoes | 90 | 76 | 1 | 3 | 0 | 0 | 9 | 1 |
| Clementine | 1 fruit | 60 | 106 | 1 | 5 | 0 | 0 | 15 | 1 |
|  |  |  |  |  |  |  |  |  |  |
| **Food items per estimated portion** | **Portion** | **Portion** | **Energy** | **Protein** | **CHO** | **Fat** | **SFA** | **Calcium** | **Fibre** |
| **Dinner** | **description** | **g** | **kJ** | **g** | **g** | **g** | **g** | **mg** | **g** |
| Spaghetti Bolognaise (Tesco Italian) | whole portion | 450 | 2556 | 43 | 55 | 23 | 10 | 108 | 6 |
| Bramley Apple Pie (Tesco) | 1 pie | 60 | 914 | 2 | 35 | 8 | 2 | 20 | 1 |
| **Alternate** |  |  |  |  |  |  |  |  |  |
| Chicken in Black Bean sauce (Tesco, no rice) | 1 pack | 318 | 1177 | 34 | 10 | 11 | 1 | 64 | 3 |
| Wholegrain rice (Uncle Bens) | half portion | 125 | 868 | 5 | 38 | 3 | 1 | 21 | 3 |
| Veg Stir fry mix (Tesco) | half pack | 170 | 214 | 2 | 7 | 1 | 0 | 73 | 4 |
| Bramley Apple Pie (Tesco) | 1 pie | 60 | 914 | 2 | 35 | 8 | 2 | 20 | 1 |
|  |  |  |  |  |  |  |  |  |  |
|  |  |  |  |  |  |  |  |  |  |
| **Food items per estimated portion** | **Portion** | **Portion** | **Energy** | **Protein** | **CHO** | **Fat** | **SFA** | **Calcium** | **Fibre** |
| **Snacks** | **description** | **g** | **kJ** | **g** | **g** | **g** | **g** | **mg** | **g** |
| Apple | 1 fruit | 133 | 301 | 1 | 16 | 0 | 0 | 7 | 2 |
| Granola bar (Nature Valley Oats and Honey) | 1 packet | 42 | 805 | 3 | 27 | 7 | 1 | 32 | 2 |
| Carr’s Crispbread multigrain | 4 crackers | 38 | 697 | 3 | 25 | 5 | 2 | 17 | 2 |
| Tomato (cherry) | 7 tomatoes | 80 | 67 | 1 | 2 | 0 | 0 | 8 | 1 |
| Ballymaloe Relish | thick spread | 15 | 102 | 0 | 6 | 0 | 0 | 6 | 0 |

**Menu 3**

| **Food items per estimated portion** | **Portion** | **Portion** | **Energy** | **Protein** | **CHO** | **Fat** | **SFA** | **Calcium** | **Fibre** |
| --- | --- | --- | --- | --- | --- | --- | --- | --- | --- |
| **Breakfast** | **description** | **g** | **kJ** | **g** | **g** | **g** | **g** | **mg** | **g** |
| Bread (Hovis best of both, toasted) | 1 slices | 40 | 397 | 4 | 16 | 1 | 0 | 191 | 2 |
| Butter (Dairygold butter sticks) | 2 pats | 14 | 417 | 0 | 0 | 11 | 7 | 3 | 0 |
| Denny traditional bacon rashers | 2 rashers | 60 | 470 | 11 | 1 | 7 | 3 | 3 | 0 |
| Orange juice (Tropicana 250ml bottles) | 1 bottle | 250 | 455 | 2 | 22 | 0 | 0 | 25 | 2 |
|  |  |  |  |  |  |  |  |  |  |
| **Food items per estimated portion** | **Portion** | **Portion** | **Energy** | **Protein** | **CHO** | **Fat** | **SFA** | **Calcium** | **Fibre** |
| **Lunch** | **description** | **g** | **kJ** | **g** | **g** | **g** | **g** | **mg** | **g** |
| Bread (Hovis best of both) | 2 slices | 80 | 794 | 8 | 33 | 2 | 0 | 382 | 3 |
| Mayonnaise (Hellmann’s) | tuna mix | 45 | 1334 | 0 | 1 | 36 | 3 | 4 | 0 |
| Tuna (Tesco, in brine) | half tin | 60 | 253 | 14 | 0 | 0 | 0 | 6 | 0 |
| Sweetcorn (Tesco natural, tinned in water) | half tin | 120 | 406 | 3 | 14 | 2 | 0 | 4 | 3 |
| Lettuce | 4 leaves | 25 | 12 | 0 | 0 | 0 | 0 | 6 | 0 |
| Tomato (cherry) | 6 tomatoes | 90 | 76 | 1 | 3 | 0 | 0 | 9 | 1 |
| Mixed berry snack pot (strw/blue) | 1 pot | 80 | 117 | 1 | 6 | 0 | 0 | 11 | 2 |
|  |  |  |  |  |  |  |  |  |  |
|  |  |  |  |  |  |  |  |  |  |
| **Food items per estimated portion** | **Portion** | **Portion** | **Energy** | **Protein** | **CHO** | **Fat** | **SFA** | **Calcium** | **Fibre** |
| **Dinner** | **description** | **g** | **kJ** | **g** | **g** | **g** | **g** | **mg** | **g** |
| Chicken Chow Mein (Tesco) | 1 pack | 320 | 1443 | 32 | 44 | 3 | 0 | 0 | 5 |
| Veg Stir fry mix (Tesco) | half pack | 170 | 214 | 2 | 7 | 1 | 0 | 73 | 4 |
| Angel Cake slices (Tesco) | 1 slice | 29 | 494 | 1 | 17 | 5 | 1 | 12 | 0 |
| **Alternate** |  |  |  |  |  |  |  |  |  |
| Spanish Paella (Birds Eye) | 1 pack | 350 | 1887 | 21 | 60 | 13 | 6 | 81 | 4 |
| Angel Cake slices (Tesco) | 1 slice | 29 | 494 | 1 | 17 | 5 | 1 | 12 | 0 |
|  |  |  |  |  |  |  |  |  |  |
| **Food items per estimated portion** | **Portion** | **Portion** | **Energy** | **Protein** | **CHO** | **Fat** | **SFA** | **Calcium** | **Fibre** |
| **Snacks** | **description** | **g** | **kJ** | **g** | **g** | **g** | **g** | **mg** | **g** |
| Clementine | 1 fruit | 60 | 106 | 1 | 5 | 0 | 0 | 15 | 1 |
| Jaffa Cakes (McVities) | 3 cakes | 37 | 587 | 2 | 26 | 3 | 2 | 17 | 1 |
| Cream crackers (Jacobs snack pack) | 4 crackers | 30 | 555 | 3 | 20 | 4 | 2 | 28 | 1 |
| Butter (Dairygold butter sticks) | 2 pats | 14 | 417 | 0 | 0 | 11 | 7 | 3 | 0 |
