# Supplementary material for: Effect of reduced-calcium and high-calcium cheddar cheese consumption on the excretion of faecal fat: a 2-week cross-over dietary intervention study
Source: Eur J Nutr. 2023 Feb 23;62(4):1755–65. doi: 10.1007/s00394-023-03118-8 (PMC10195742; doi:10.1007/s00394-023-03118-8)
Supplement: Supplementary file 2 — Supplementary file2 (DOCX 19 KB) [file 394_2023_3118_MOESM2_ESM.docx]

**Table S2. Fatty acid content (as a percent of the total fat) across dietary intervention periods**

| Fatty Acid | | HCC | | RCC | | RCC+Supp | |  |
| --- | --- | --- | --- | --- | --- | --- | --- | --- |
| Common name | Formula | Mean | SD | Mean | SD | Mean | SD | *P* |
| Butyric acid | C4:0 | 0.31 | 0.39 | 0.27 | 0.37 | 0.19 | 0.15 | 0.764 |
| Caproic acid | C6:0 | 0.12 | 0.22 | 0.07 | 0.13 | 0.04 | 0.04 | 0.645 |
| Caprylic acid | C8:0 | 0.09 | 0.09 | 0.06 | 0.05 | 0.07 | 0.13 | 0.868 |
| Capric acid | C10:0 | 0.16 | 0.09 | 0.12 | 0.05 | 0.16 | 0.13 | 0.629 |
| Undecylic acid | C11:0 | 0.01 | 0.01 | 0.02 | 0.01 | 0.01 | 0.00 | 0.669 |
| Lauric acid | C12:0 | 0.95 | 0.68 | 0.77 | 0.65 | 1.18 | 1.40 | 0.738 |
| Tridecylic acid | C13:0 | 0.04 | 0.01 | 0.04 | 0.01 | 0.04 | 0.01 | 0.958 |
| Myristic acid | C14:0 | 3.43 | 1.03 | 3.03 | 0.97 | 3.76 | 1.29 | 0.484 |
| Myristovaccenic acid | C14:1 | 0.13 | 0.03 | 0.13 | 0.05 | 0.13 | 0.05 | 0.825 |
| Pentadecylic acid | C15:0 | 1.34 | 0.26 | 1.44 | 0.29 | 1.35 | 0.33 | 0.789 |
| Palmitic acid | C16:0 | 36.75 | 7.19 | 34.86 | 6.34 | 38.61 | 4.64 | 0.533 |
| Hexadecenoic Acid | C16:1w9 | 0.06 | 0.02 | 0.07 | 0.03 | 0.05 | 0.02 | 0.278 |
| Palmitoleic Acid | C16:1w7 | 0.34 | 0.12 | 0.42 | 0.15 | 0.38 | 0.15 | 0.602 |
| Heptadecenoic acid | C17:1 | 0.04 | 0.01 | 0.04 | 0.01 | 0.04 | 0.02 | 1 |
| Stearic acid | C18:0 | 25.58 | 5.41 | 24.24 | 3.97 | 27.64 | 5.87 | 0.476 |
| Oleic acid | C18:1w9 | 15.64 | 4.38 | 15.78 | 3.77 | 12.92 | 1.63 | 0.248 |
| Vaccenic acid | C18:1w7 | 5.00 | 1.10 | 5.58 | 1.68 | 5.07 | 1.04 | 0.667 |
| Trans Vaccenic acid | C18:1w7 | 0.19 | 0.08 | 0.18 | 0.08 | 0.19 | 0.08 | 0.925 |
| Linoleic acid | C18:2w6 | 6.86 | 1.34 | 8.75 | 3.59 | 4.50 | 1.24 | 0.011* |
| y-linolenic acid | C18:3w6 | 0.19 | 0.03 | 0.21 | 0.03 | 0.18 | 0.02 | 0.29 |
| a-linolenic acid | C18:3w3 | 0.48 | 0.18 | 0.65 | 0.43 | 0.38 | 0.13 | 0.217 |
| CLA | cis9_t11 | 0.20 | 0.14 | 0.19 | 0.11 | 0.16 | 0.05 | 0.705 |
| Stearidonic acid | C18:4w3 | 0.00 | 0.01 | 0.00 | 0.01 | 0.00 | 0.00 | 0.84 |
| CLA | Perc_t10_cis12 | 0.02 | 0.01 | 0.02 | 0.02 | 0.01 | 0.01 | 0.7 |
| Arachidic acid | C20:0 | 0.77 | 0.20 | 0.77 | 0.13 | 0.81 | 0.19 | 0.901 |
| Gondoic acid | C20:1w9 | 0.26 | 0.06 | 0.31 | 0.08 | 0.27 | 0.10 | 0.485 |
| Eicosadienoate | C20:2w6 | 0.08 | 0.10 | 0.10 | 0.10 | 0.08 | 0.06 | 0.921 |
| Dihomo-y-linolenic acid | C20:3w6 | 0.06 | 0.07 | 0.08 | 0.07 | 0.06 | 0.03 | 0.854 |
| Arachidonic acid | C20:4w6 | 0.11 | 0.04 | 0.15 | 0.06 | 0.12 | 0.04 | 0.34 |
| Eicosatrienoic | C20:3w3 | 0.01 | 0.01 | 0.00 | 0.01 | 0.01 | 0.01 | 0.69 |
|  | C20:5wC3 | 0.00 | 0.00 | 0.00 | 0.01 | 0.00 | 0.00 | 0.86 |
| Behenic acid | C22:0 | 0.59 | 0.29 | 0.59 | 0.09 | 0.59 | 0.10 | 1.00 |
|  | C22:1w11 | 0.01 | 0.01 | 0.01 | 0.01 | 0.01 | 0.01 | 0.69 |
|  | C22:1w9 | 0.17 | 0.18 | 0.24 | 0.24 | 0.26 | 0.24 | 0.74 |
|  | C22:5w6 | 0.05 | 0.03 | 0.07 | 0.04 | 0.07 | 0.04 | 0.65 |
|  | C22:5w3 | 0.03 | 0.02 | 0.04 | 0.03 | 0.03 | 0.01 | 0.41 |
| Lignoceric acid | C24:0 | 0.44 | 0.19 | 0.43 | 0.06 | 0.39 | 0.07 | 0.75 |
| Cervonic Acid | C22:6w3 | 0.11 | 0.07 | 0.14 | 0.16 | 0.07 | 0.08 | 0.53 |
| Nervonic Acid | C24:1 | 0.10 | 0.03 | 0.12 | 0.03 | 0.13 | 0.07 | 0.54 |

Dietary intervention periods are: HCC (high calcium cheese), RCC (Reduced calcium cheese), and RCC + Supplement (Reduced calcium cheese + CaCO_3_ supplement). *P* refers to the overall effect of treatment in an ANOVA model in which the value obtained after each period was modeled as the dependent variable, and treatment modeled as the fixed variable. Including body weight as a co-variate did not change the results and since the values were a % of the total fat it was not included in this model. **P*-value is NS after Bonferroni correction
